# Population-based study on the prevalence of and risk factors for human papillomavirus infection in Qujing of Yunnan province, Southwest China

**DOI:** 10.1186/1743-422X-9-153

**Published:** 2012-08-08

**Authors:** Sun Lu-lu, Jin Qiong, Li Hui, Zhou Xian-rong, Song Zhi-qin, Cheng Xue-mei, Tao Tao, Liang Bing, Xu Lin, Wang Yu-rong, Zhen Yan, He Ji-wen, Shen Keng

**Affiliations:** 1 Department of Obstetrics and Gynecology, Peking Union Medical College Hospital, Peking Union Medical College/Chinese Academy of Medical Sciences, Beijing 100730, China; 2 Department of Epidemiology, Institute of Basic Medical Sciences, Peking Union Medical College/Chinese Academy of Medical Sciences, Beijing 100730, China; 3 Obstetrics and Gynecology Hospital of Fudan University, Shanghai 200011, China; 4 Department of Obstetrics and Gynecology, PLA Navy General Hospital, Beijing 100000, China; 5 Department of Obstetrics and Gynecology, The first hospital of Kunming University, Yunnan 650032, China; 6 Department of Obstetrics and Gynecology, Qujing Maternal and Child Care Service Centre, Yunnan 655000, China; 7 Vice Chairman of the National Women's Federation, Beijing 100730, China; 8 Director of Qujing Health Care Bureau, Qujing 655000, China

**Keywords:** Human papillomavirus, Genotype, Cervix, Epidemiology, China

## Abstract

**Background:**

Human papillomavirus (HPV) infection causes cervical cancer and premalignant lesions of the cervix. Prevalence of HPV infection and HPV genotypes vary among different regions. However there is no data on the prevalence of HPV infection and HPV genotypes from southwest China. This study was undertaken to determine the prevalence of and risk factors for HR-HPV infection in Qujing of Yunnan province, southwest China to provide comprehensive baseline data for future screening strategies.

**Methods:**

A sample of 5936 women was chosen by the multi-stage stratified cluster sampling method with selection probabilities proportional to size (PPS). An epidemiological questionnaire was conducted via a face-to-face interview and cervical specimens were taken for HPV DNA testing by Digene Hybrid Capture 2 (HC2) test. HPV Genotyping Reverse Hybridization Test was used for HPV genotyping. Proportions were compared by Chi-squared tests, and logistic regression was utilized to evaluate risk factors.

**Results:**

The median age was 38 years and the inter-quartile range was from 31 years to 47 years. 97.3% of the study population was Han nationality. Overall prevalence of HR-HPV infection was 8.3% (494/5936) and bimodal age distribution of HPV infection was observed. The five most prevalent HR-HPV genotypes were HPV-16(3.4%), HPV-56(1.7%), HPV-58(1.4%), HPV-33(1.2%) and HPV-52(0.88%). Multiple HPV infections were identified in 50.5% (208/412) of the positive genotyping specimens. Multivariate logistic regression model indicated that parity (OR = 1.35, 95% CI: 1.18-1.53, p < 0.0001) was a risk factor for HR-HPV infection, and age of 50–65 years (OR = 0.60, 95% CI: 0.45-0.80, p = 0.0005), being married or in stable relationship (OR = 0.55, 95% CI: 0.31-0.96, p = 0.035) were protective factors.

**Conclusions:**

This study provided baseline data on HR-HPV prevalence in the general female population in Qujing of Yunnan province, southwest China. The finding of multiple HPV infections and bimodal age distribution revealed that HPV screening is necessary for perimenopausal women in future.

## Background

Cervical cancer is the second most common cancer in women worldwide. [1] More than 100 HPV types have been identified and nearly 40 types are known to infect the genital tract [2]. HPV 16 and 18 have been shown to be causative in the etiology of cervical cancer [3]. Women with persistent infection with oncogenic HPV subtypes may develop cervical cancer.

There are about 20 high-risk subtypes of HPV associated with cervical cancer and precancerous lesions. [3] Overall, HPV-16 and HPV-18 account for approximately 70% of all cervical cancer diagnosed worldwide each year [4]. HPV genotypes differ greatly in their geographic distribution [5,6]. However, there is no data on the prevalence of HPV infection from southwest China. Qujing located at an altitude of 2000 meters in southwest China. As the second largest city of Yunnan province, the population is 6.16 million, and there are various ethnic groups. This study seeks to describe the prevalence of HPV infection, HPV genotype distribution as well as risk factors for HR-HPV infection in the general female population of Qujing, Yunnan province, southwest China to provide comprehensive baseline data for assessing its potential impact on cervical pathology and future screening strategies of cervical cancer in China.

## Methods

### Study population and survey sampling design

This cross-sectional population-based study was conducted in Qujing City during June to July 2010. The study population comprised all resident women between the ages of 18 to 65 years. The sample size was calculated on the basis of an expected prevalence of HPV infection, derived from previous studies [7,8]. The formula was as following:

$$n=\left(\frac{{u_{\alpha }}^{2}\times \pi \times \left(1-\pi \right)}{\delta^{2}}\right)$$

where,

n_0_: estimated sample size;

*π*: expected prevalence of HPV infection, 5% was used;

*δ*: allowing within 15% of the range for a prevalence of 5%, *δ* = 0.15*π*;

*α* = 0.05, *u*_*α*_ = 1.96.

In the expectation of a response rate of 80% and expanded sample size of 150%, the sample size was adjusted using n = 1.5n_0_: 0.80 = 6083. The estimated sample size for the survey was 6100 rounded to the nearest hundred. Non-contactables were defined as individuals who were not contactable on three occasions, and refusals defined as individuals who declined to participate in the study. And women who had a hysterectomy, were menstruating or pregnant at the time of the study were excluded. Sample size supplement is not necessary if non-contactables and refusals account for less than 20%.

**Figure 1:**
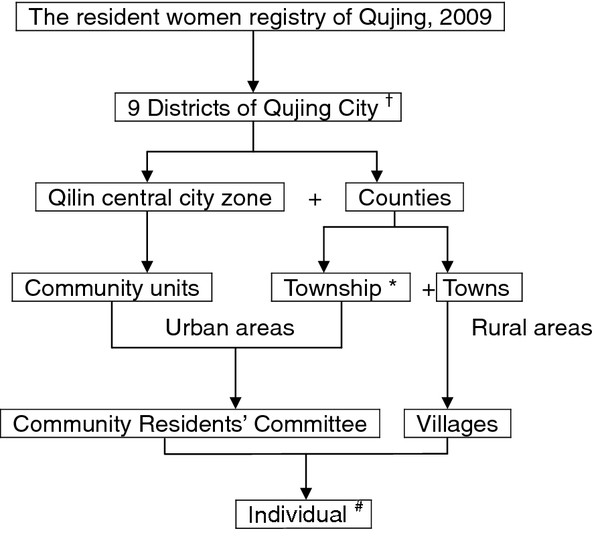
**Strategy of sampling.**^†^ Qilin central city zone, Xuanwei city, and 7 counties. * The town where the local government of the county located. ^#^ Individuals were drawn up with simple random sampling method. 9 age strata with PPS were defined in order to maintain a large enough sample size for statistical power.

A multi-stage stratified cluster sampling method with selection probabilities proportional to size (PPS) was designed to recruit participants, based on the resident women registry of Qujing, 2009. The stratification factors included geographical area and age. Qujing City was comprised of 9 districts, including Qilin central city zone, Xuanwei city, and 7 counties. Urban area was defined as community units of Qilin and townships (the town where the local government of the county located) of the counties. Rural area was defined as rural villages of counties. In order to maintain a large enough sample size for statistical power within each strata, 9 age strata with the proportion to size were defined. The sampling strategy is shown in Figure 1.

In the first stage, 4 districts were taken from the 9 districts of Qujing, including Qilin central city zone and 3 counties of Huize, Zhanyi and Luliang. In the second stage, 3 community units from Qilin, one township and 4 towns from each county were taken with probability proportional to size. The third stage involved sampling of 6 communities within each community units and 3 villages within each town. Therefore, 72 study sites and 85 individuals from each site were recruited in the survey. Finally, 85 eligible women provided 9 age strata from each selected area were drawn up with simple random sampling method, based on the local resident women registry. The four-stage stratified cluster sampling method is shown in Table 1.

**Table 1:** Multi-stage stratified cluster sampling method

	**Stage 1**	**Stage 2**	**Stage 3**	**Stage 4**	**Sample size**
Urban areas	Central city zone	Community units / Township	Community Residents’ Committee	Individuals	
	×1	×6	×6	×85	3060
Rural areas	Counties	Towns	Villages	Individuals	
	×3	×4	×3	×85	3060

### Questionnaire-based interview

Verbal informed consent was obtained before the questionnaire interview. Participants were asked to complete a questionnaire via a face-to-face interview, which was designed to elicit socio-demographic status, sexual behavior, history of sexually transmitted infection (STI), parity, and other probable risk factors for cervical cancer. The questionnaire was piloted on 85 outpatients from gynecological clinic in Peking Union Medical College Hospital (PUMCH). The internal consistency reliability (Cronbach’s α) for the full scale was 0.825. Furthermore, face validity and content validity of the questionnaire were both confirmed by epidemiologists and experts from Department of Obstetrics and Gynecology, PUMCH. The results indicated that the questionnaire had good reliability and validity.

### Cervical specimen and HR-HPV detection

Informed consent was obtained before the collection of the cervical specimens. Samples of exfoliated cervical cells were collected with DNAPap cervical sampler brush (Digene) during gynecological examinations. The sampler was inserted 1–1.5 cm into the endocervical canal and rotated 3–5 full turns in counterclockwise direction. The cervical sampler was then placed into transport medium and stored at 4°C. HR-HPV detection was carried out using the Digene Hybrid Capture 2 ® (HC2) test according to the manufacture’s instructions, as described previously. The results are given as a relative light unit (RLU) ratio and a positive result was defined as RLU/Co ≥ 1.00 (5000 copies of HPV genome).

### HPV genotyping

Genotyping was carried out by HPV Genotyping Reverse Hybridization Test RUO detection kit. Phenol-chloroform was used to extract HPV DNA from the residual of HC2 positive samples. And the human β-globin gene was used as internal control (IC) to verify the quality for polymerase chain reaction (PCR). DNA of specimens with negative internal control amplification result were re-extracted and re-amplified. GP5+/6+ consensus primers were used according to the manufacture’s instructions [9].

HPV Genotyping Reverse Hybridization Test identifies 18 high risk or probably high risk HPV genotypes. Besides 13 HR-HPV genotypes covered by HC2 (including HPV-16, 18, 31, 33, 35, 39, 45, 51, 52, 56, 58, 59 and 68), there are 5 other HPV genotypes that are not included in the current HC2 probe cocktail (including HPV-26, 53, 66, 73 and 82) [10].

The final results were detected by colorimetric change on the membrane strip immortalized with type-specific oligonucleotides probes. All procedures were carried out according to the manufacture's instructions. HR-HPV positive cases were defined as those with single-type (a single HR type) infection and those with multiple-type (two or more HR types) infection. Type-specific prevalence included that in both single-type and multiple-type infection [11].

### Quality assurance and quality control

Several efforts were made to standardize all procedures in each study site. Manuals were written with instructions on how to conduct the fieldwork, interviewing, collecting cervical samples, processing and storing the samples collected. The training program for doctors and crew of local stuff was held a week before investigation. Data were key-entered twice by two experienced typists. Independent databases were formed and were compared with each other. Any mismatch was corrected after inter-comparison of the databases and a proportion of 10% of the records in the final database were drawn up randomly for re-correction manually.

For the laboratory tests, HPV DNA testing by the HC2 was conducted using the same commercial kit and the same machine in local lab of Qujing. Cervical samples were transported to central lab in Shenzhen for HPV genotyping thereafter. All of the laboratory technicians were blinded to the clinical profile of the clients. HPV genotyping were independent of HPV DNA tests.

Ethical approval was granted by the Research Ethics Committee of Peking Union Medical College Hospital, Peking Union Medical College & the Chinese Academy of Medical Sciences.

### Statistical analysis

Descriptive statistics (medians and proportions) were utilized to characterize the variables. Proportions were compared using Chi-squared tests. Odds ratios (ORs) with 95% confidence intervals (CIs) were calculated using unconditional logistic regression (Mantel-Haenszel analysis) to estimate independent risk factors for HR-HPV infection. Age and variables associated with HR-HPV infection on univariate logistic regression model were included as candidates in multivariate model. Parity, age of 50–65 years and marital status were included in the final model by stepwise selection.

Data analysis was conducted using the SAS System for Windows (SAS Institute Inc., USA). A level of 0.05 was chosen to indicate statistical significance.

## Results

Qujing is the second largest city of Yunnan province in southwestern China, with an average altitude of 2000 m. It has a population of 6 million and has eight ethnic groups. Because of the limited medical resources, cervical cancer screening is not widely utilized, especially in rural areas of Qujing. Of the 6116 women who participated in the study, 180 were excluded because of inadequate cytology specimen, negative β-globin or inadequate questionnaire information, leaving a total of 5936 women included in the statistical analyses. The median age was 38 years and the inter-quartile range was from 31 years to 47 years. 97.3% of the study population was Han nationality.

### HR-HPV prevalence

**Figure 2:**
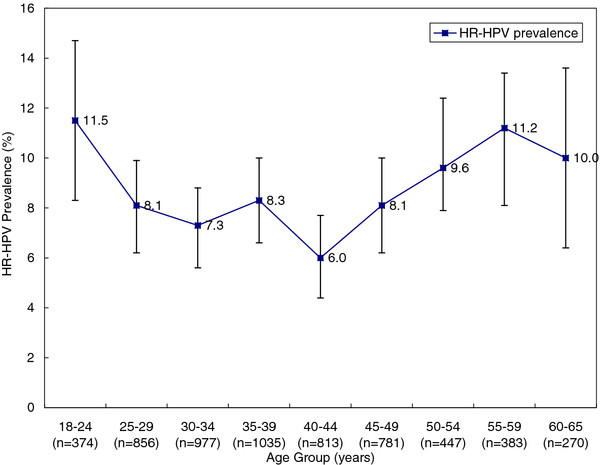
Age-specific prevalence for HR-HPV infection and 95% CI.

Of the 5936 detected cervical samples, 494(8.3%) were HR-HPV positive by the HC2 test. The prevalence of HR-HPV ranged from 11.5% (95% CI: 8.3%-14.7%) among women aged 18–24 years to 6.0% (95% CI: 4.4%-7.7%) among women aged 40–44 years. Nevertheless, a relatively high prevalence of 11.2% (95% CI:8.1%-14.4%) for HR-HPV among women aged 55–59 was observed (*χ*^2^ = 18.343,*P* = 0.019). Figure 2 shows the age-specific prevalence for HR-HPV infection.

### HPV genotypes

Among the 494 HPV positive samples, there were 412 samples infected by any type of HPV identified by HPV Genotyping, including 15 different HR-HPV genotypes. The five most prevalent types were HPV-16(3.4%), HPV-56(1.7%), HPV-58(1.4%), HPV-33(1.2%) and HPV-52(0.88%), accounted for 80.1% of all HR-HPV types detected (Table 2). When stratified by geographical area, the most prevalent types in urban areas were HPV-16(3.2%), HPV-56(1.5%), HPV-58(1.2%), HPV-33(1.0%) and HPV-18(0.75%); the most prevalent types in rural areas were HPV-16(3.6%), HPV-56(1.8%),HPV-58(1.6%), HPV-33(1.4%) and HPV-52(0.98%). Multiple HPV infections were identified in 50.5% (208/412) of the genotyping positive specimens, and two peaks of multiple HPV infections were detected at 18–24 and 45–54 years age groups respectively. The most common combination of multiple HPV infections was HPV-16 and −56 (50/208) followed by HPV-16 and −58 (44/208). Of multiple HPV infections, 58.7% (122/208) were infected with two types, 26.0% (54/208) were infected with three types, whereas 15.3% (32/208) were infected with more than four HPV types.

**Table 2:** HPV prevalence for specific types, both overall and by age group (n = 5936) *

**HPV type**	**No**	**Overall prevalence,%**	**Prevalence by age group,%**								
			**18-24 (n = 374)**	**25-29 (n = 856)**	**30-34 (n = 977)**	**35-39 (n = 1035)**	**40-44 (n = 813)**	**45-49 (n = 781)**	**50-54 (n = 447)**	**55-59 (n = 383)**	**60-65 (n = 270)**
Any HR types	412	6.9	9.4	7.0	5.0	6.9	5.4	7.7	8.7	8.1	8.5
16	203	3.4	2.2	4.4	2.4	4.0	3.2	1.8	5.0	3.2	3.8
18	45	0.8	0.5	0.7	1.0	1.0	0.4	0.6	2.0	0	0
26	4	0.07	0	0.1	0	0	0	0.3	0.1	0	0
31	40	0.7	1.1	0.9	0	0.7	1.2	0.9	0	0.8	0.7
33	72	1.2	2.4	0.7	0.6	0.8	0.7	1.2	0	5.0	3.3
35	9	0.15	0	0.1	0.1	0.4	0.2	0.1	0	0	0
39	44	0.74	2.1	1.6	0.3	0.5	0.4	0.4	1.1	0.8	0
45	15	0.25	0.5	0	0.2	0	0.6	0.3	0	0.5	0.7
51	17	0.29	0.3	0.1	0.2	0.4	0.4	0.4	0.4	0.3	0
52	52	0.88	1.6	0.8	0.6	1.1	0.7	0.6	1.1	1.0	0.7
56	103	1.7	1.1	1.2	2.6	1.5	1.0	2.2	1.3	2.1	3.3
58	84	1.4	1.6	2.3	0.6	1.0	1.0	1.5	2.0	0.8	3.7
59	7	0.12	0.3	0	0.2	0.1	0	0	0.4	0	0.4
66	31	0.52	1.3	0.5	0.6	0.3	0.1	0.5	1.1	0.5	0.4
68	19	0.32	0	0.4	0.3	0.4	0.1	0.6	0.2	0.5	0
Single	204	3.4	3.5	4.6	2.9	3.7	2.8	2.9	2.9	3.9	4.4
Multiple	208	3.5	5.9	2.5	2.1	3.2	2.6	4.7	5.8	4.2	4.1

HPV: human papillomavirus, No.: number, HR: high-risk.

*The prevalence of an individual type or category included detection of this type or category in both single- and multiple-type infection.

### Risk profile for HR-HPV infection

The socio-demographic and reproductive risk factors for HR-HPV infection are presented in Table 3 with age-adjusted odds ratios (OR) and 95% confidence intervals (CI). On univariate logistic regression, women aged 30–39 years and women living in an urban area were at increased risk for HR-HPV infection (OR = 1.30 and 1.31; p = 0.005). Being married or in a stable relationship, and having an average personal family income above 4000¥ (OR = 0.37 and 0.78; p < 0.0001 and p = 0.013) were found to be protective factors for HR-HPV infection.

**Table 3:** Major risk factors for HR-HPV infection adjusted for age

**Characteristic**	**Overall No.**^**†**^	**HPV DNA positive No. (%)**	**P value**	**OR (95%CI)**
Age (years)			0.72	
18-29	1230	112 (9.1)		0.75 (0.49-1.15)
30-39	2012	157 (7.8)		1.30 (1.09-1.58)
40-49	1594	112 (7.0)		1 (Ref)
50-65	1100	113 (10.3)		0.58 (0.41-0.81)
Race				
The Han nationality	5501	461 (8.4)		1 (Ref)
Minority	155	15 (9.7)	0.566	0.86 (0.50-1.48)
Region				
Urban areas	2977	276 (9.3)	0.005	1.31 (1.09-1.58)
Rural areas	2959	218 (7.4)		1 (Ref)
Income per year				
<4000¥	2761	209 (7.6)		1 (Ref)
≥4000¥	2781	258 (9.3)	0.013	0.78 (0.65-0.95)
Marital status				
Married or in stable relationship	5471	449 (8.2)	<0.0001	0.37 (0.23-0.58)
Unmarried*	159	24 (15.1)		1 (Ref)
Education				
Primary/Junior school	4196	344 (8.2)		1 (Ref)
High school/University	1471	132 (9.0)	0.300	0.89 (0.72-1.11)
Smoking				
Ever smoked	207	16 ()	0.731	0.91 (0.54-1.53)
Never	5379	450 ()		1 (Ref)
Age at first intercourse				
≤17	41	16 (7.8)		1 (Ref)
≥18	5599	459 (8.2)	0.100	1.39 (0.81-2.38)
Age of menarche				
≤12	407	34 (8.4)		1 (Ref)
≥13	5257	444 (8.4)	0.961	1.01 (0.70-1.46)
Delivery				
≤2	4533	381 (8.4)		1 (Ref)
≥3	702	52 (7.4)	0.372	1.26 (0.92-1.73)
Oral contraceptive pills				
Never	5486	456 (8.3)	0.22	1.48 (0.82-1.66)
Ever	110	13 (11.8)		1 (Ref)
Life time sexual partners				
1	4924	407 (8.3)		1 (Ref)
≥2	57	6 (10.5)	0.470	0.77 (0.33-1.80)
Husband’s life time sexual partners				
1	5114	422 (8.3)		1 (Ref)
≥2	107	14 (13.1)	0.078	1.67 (0.95-2.96)
Menopause				
Yes	867	91 (10.5)		1 (Ref)
No	4882	391 (8.0)	0.325	1.23 (0.82-1.83)

† Numbers did not always add up to total due to missing data.

* Unmarried includes single, divorced, separated and widowed.

OR = odds ratio; CI = confidence interval.

In multivariate model, all variable from the univariate model were included. Results of the regression model are shown in Table 4. In the final model, parity was a risk factor for HR-HPV infection (OR = 1.35, 95% CI: 1.18-1.53, *P* < 0.0001), and age of 50–65 years (OR = 0.60, 95% CI: 0.45-0.80; *P* = 0.0005), married or in stable relationship (OR = 0.55, 95% CI: 0.31-0.96, *P* = 0.035) were protective factors for HR-HPV infection.

**Table 4:** Summary of the final multivariate model (Stepwise Selection, slentry = 0.5, slstay = 0.05)

**Variables**	***β***	***β***_**SE**_	**Chi-Square**	***p***	**OR**	**95%CI**
Intercept	1.9918	0.1214	269.0788	<.0001	-	-
Parity	0.2976	0.0664	20.0954	<.0001	1.347	1.182 ~ 1.534
50-65 years old	−0.5169	0.1479	12.2244	0.0005	0.596	0.446 ~ 0.797
Being married or in stable relationship	−0.6044	0.2862	4.4586	0.0347	0.546	0.312 ~ 0.958

## Discussion

Differences in HR-HPV prevalence are seen between countries. Previous studies have documented that HR-HPV prevalence in women attending routine cervical screening program in Europe was 15.6%. It was 13-18% in Latin America, 17% in India and 26% in Nigeria [5], which were much higher than the prevalence of this study in China. To date, there has been no population-based data in the prevalence of HR-HPV infection in southwestern China. The present study demonstrated that HR-HPV infection in the general female population in Qujing is 8.3%. Compared with other studies, HR-HPV infection in our study population is lower than that in central China of Shanxi (12.2%), northeast China of Shenyang (11.7%), southeast China of Zhejiang (10.2%), and southeastern coast China of Shenzhen (13.5%) and Taiwan (32.4%) [12-17]. However some studies that reported even lower HR-HPV infection are the ones in Beijing (5.8%) and our previous study in Tibet (7.1%) [7,8]. HR-HPV infection rate may vary among different regions because of different culture and different life styles. One interpretation attributed to the relatively lower prevalence of HPV infection in this study may be that Qujing located in a close mountainous area keeping with the traditional Chinese social habits and life styles. Stable marital status and sexual partner may provide a protection for HPV infection [18-22].

The persistence and clearance of HPV infection result in the actual HPV infection rate. [23,24] Bimodal age distribution of HR-HPV is also observed in this study. The first peak of HR-HPV infection is obvious in women aged 18–24 years, which may be due to primary exposure to HPV after sexual initiation and lack of adaptive immune responses in young women. [25-27] The second peak of HR-HPV infection is observed in women aged 55–59 years which should be deserved more attention. It is assumed that the immunologic and physiologic deregulation caused by hormone fluctuations may explain the high infection rate around menopausal women [28,29]. Nevertheless, the positive HR-HPV infection around menopausal period women may imply the persistent HR-HPV infection [28,30,31]. Therefore, HPV detection is clinically valuable for perimenopausal women in cervical cancer screening program. For perimenopausal women with positive HR-HPV DNA test result, regular follow-up and standard management should be considered, including repeated cytology and colposcopy if ASCUS or more severe cytological findings were reported, because they have higher risk for the development of cervical cancer [18]. However, because of regional economy imbalance in China, HPV testing is currently not available in some regions, especially in west China.

The five most common HPV types in this study were HPV-16, -56, -58, -33 and −52 respectively. Some studies had shown that HPV-52 and −58 were more predominant and overrepresented in cervical cancer cases in Asia [5,12,13,15,17,32]. Data of this study revealed that HPV-16,-56, -58, -33 and −52 may play a role in the etiopathology of cervical cancer. Meanwhile, the high prevalence of multiple HPV infection in this study is also detected in young women with sexual activity as well as in women around menopausal period. Some researches have reported that women with multiple HPV infection were at increased risk of precancerous lesions. [33,34] Our previous study in Tibet revealed that significant difference was evident when comparing the percentage of abnormal cytological results in multiple HPV infection with single HPV infection [8]. Therefore, the role of multiple HPV infection in etiology of cervical cancer should be further investigated, especially for the research on HPV vaccine.

HPV vaccines have shown type-restricted prophylactic efficacy for genital lesions. However, it does not have clinical significance for women who have been infected with HPV. As a result, HPV vaccine program will be of greater benefit to younger women. We assume that the HPV prophylactic vaccines including HPV-52 and −58 may offer higher protection for young women in China and other Asian countries.

## Conclusions

This study is the first report on HR-HPV prevalence, HPV genotypes as well as the risk factors for HR-HPV infection in the general female population in southwestern China. The baseline data of this study will contribute to making strategies for women’s health care and implementation of cervical cancer screening program in China.

## Abbreviations

HPV: Human papillomavirus; HR: High-risk; LR: Low-risk; PCR: Polymerase chain reaction; OR: Odds ratio; CI: Confidence interval.

## Competing interests

The authors declare that they have no competing interests.

## Authors' contributions

SLL participated in the study design, carried out the study, collected data, performed analysis of data and drafted the manuscript. JQ participated in the study design, developed the assay protocol, carried out the study and coordination. LH contributed to study design and provided consultation. ZXR, SZQ, XL participated in the training program, quality control and coordination. TT, LB, WYR carried out the study and quality control. CXM provided consultation and quality control. ZY, HJW carried out the study, data collection and quality control. SK participated in the study design, conceived the study, provided consultation, coordination and revised the manuscript. All authors have read and approved the final manuscript.
